# Genome sequences characterizing five mutations in RNA polymerase and major capsid of phages ϕA318 and ϕAs51 of *Vibrio alginolyticus* with different burst efficiencies

**DOI:** 10.1186/1471-2164-15-505

**Published:** 2014-06-21

**Authors:** Wangta Liu, Ying-Rong Lin, Ming-Wei Lu, Ping-Jyun Sung, Wei-Hsien Wang, Chan-Shing Lin

**Affiliations:** Department of Marine Biotechnology and Resources, Asia-Pacific Ocean Research Center, National Sun Yat-sen University, Kaohsiung, 80424 Taiwan; Department of Biotechnology, Kaohsiung Medical University, Kaohsiung, Taiwan; Department of Aquaculture, National Taiwan Ocean University, Keelung, Taiwan; National Museum of Marine Biology and Aquarium, Pingtung, 944 Taiwan

**Keywords:** RNA polymerase truncation, Spine Helix, Mutation in F-loop

## Abstract

**Background:**

The burst size of a phage is important prior to phage therapy and probiotic usage. The efficiency for a phage to burst its host bacterium can result from molecular domino effects of the phage gene expressions which dominate to control host machinery after infection. We found two *Podoviridae* phages, ϕA318 and ϕAs51, burst a common host *V. alginolyticus* with different efficiencies of 72 and 10 PFU/bacterium, respectively. Presumably, the genome sequences can be compared to explain their differences in burst sizes.

**Results:**

Among genes in 42.5 kb genomes with a GC content of 43.5%, 16 out of 47 open-reading frames (ORFs) were annotated to known functions, including RNA polymerase (RNAP) and phage structure proteins. 11 strong phage promoters and three terminators were found. The consensus sequence for the new vibriophage promoters is AATAA*AGT****T****G*CCCTATA, where the *AGT****T****G* bases of −8 through −12 are important for the vibriophage specificity, especially a consensus ***T*** at −9 position eliminating RNAP of K1E, T7 and SP6 phages to transcribe the genes. ϕA318 and ϕAs51 RNAP shared their own specific promoters. In comparing ϕAs51 with ϕA318 genomes, only two nucleotides were deleted in the RNAP gene and three mutating nucleotides were found in the major capsid genes.

**Conclusion:**

Subtle analyses on the residue alterations uncovered the effects of five nucleotide mutations on the functions of the RNAP and capsid proteins, which account for the host-bursting efficiency. The deletion of two nucleotides in RNAP gene truncates the primary translation due to early stop codon, while a second translational peptide starting from GTG just at deletion point can remediate the polymerase activity. Out of three nucleotide mutations in major capsid gene, H53N mutation weakens the subunit assembly between capsomeres for the phage head; E313K reduces the fold binding between β-sheet and Spine Helix inside the peptide.

**Electronic supplementary material:**

The online version of this article (doi:10.1186/1471-2164-15-505) contains supplementary material, which is available to authorized users.

## Background

*Vibrio* strains are reported to be fatal pathogens not only to humans but also to aquaculture fish and shellfish. Several virulence factors that they produce—including hemolysin, caseinase, gelatinase, lipase, and phospholipase—are detrimental to animal health [1–4]. For instance, an extracellular protease with endopeptidolytic and exopeptidolytic activities from a pathogenic *V. pelagius* strain is responsible for the lethal effect and vibriosis in turbot. The different virulence factors are produced at different stages of infection. Hemolysin-producing bacteria include *V. parahaemolyticus*, *V. alginolyticus*, *V. cholerae*, *V. hollisae*, *Aeromonas veronii*, and *V. anguillarum* (*Listonella anguillarum*).

*Vibrio alginolyticus*, a Gram-negative halophilic bacterium that frequently occurs in the normal microbiota of marine environments, is a pathogen of epizootic outbreaks, which causes serious mortality of fish and shellfish and also can transmit to humans. The disease caused by *V. alginolyticus* often has lethal consequences for fish larvae throughout the world, and it has become one of the major limiting factors in aquaculture in developing countries. For example, it was a causal agent of vibriosis outbreaks in grouper sea bream *Sparus aurata* [2]. *V. alginolyticus* causes several symptoms in sea bream: septicemia, hemorrhaging, dark skin, and ulcers on the skin surface. Some fish accumulate fluid in the peritoneal cavity or have hemorrhagic livers. In several cases of high-mortality outbreaks, *Vibrio alginolyticus* and *Vibrio splendidus* biovar II were the primary organisms found in moribund clam larvae (*Ruditapes decussatus*), and these two species processed extracellular products collaboratively at high concentrations to kill the hemocytes after four hours of incubation. Neurotoxic effects caused by the supernates from diseased strains of *Vibrio alginolyticus* and *Vibrio anguillarum* caused several symptoms in trout, including convulsions, wriggling, contortive swimming and respiratory arrest [2].

Chemotherapeutic agents are effective to control pathogenic bacteria in aquaculture. Nevertheless, emergence of antibiotic-resistant bacteria has become a critical issue [3]. As a result of management practices in production cycles, the use of probiotics to prevent pathogenic bacterium growth in aquaculture is a practice to reduce both issues on antibiotic resistance and drug residues in food. One alternative is to use bacteriophages to control bacterial growth, which is similar to phage therapy in medical care. This idea has been proposed as a cure for coral disease [5]. A variety of bacteriophages that can quickly lyse the pathogens have high potential for future applications in aquaculture.

The burst size of a phage represents an overall outcome that is so important for phage therapy and probiotic usage. The molecular interactions between phage and its host under certain environmental conditions are complicated to resolve. Sequences of bacteriophage genomes provide a magnificent tool to investigate interactions with hosts. In addition, vibriophages can be used as a new typing scheme for pathogen identification and as candidate agents for phage therapy and probiotics. For example, the roles of lytic bacteriophages in cholera epidemics were investigated, such as filamentous CTXϕ, myovirus ICP1, and podovirus ICP3 of *Vibrio cholerae*, causing infant and adult deaths for centuries [6]. Though quite a large number of *Vibrio* phages have been described, only a few of complete genome sequences are known. Genome sequences of lytic vibriophages are new keys for molecular typing, pathogenesis, and even assessment of therapeutic efficacy. Complete genomes of some phages of *V. cholerae*, *V. parahaemolyticus*, and *V. vulnificus* were documented, yet no genome for *V. alginolyticus* podovirus has been reported. In this study, we characterized the genome sequences of two newly-isolated phages that can lyse *V. alginolyticus* ATCC 17749 with different efficiencies.

## Results

### Host range, burst size, and thermal stability

**Figure 1:**
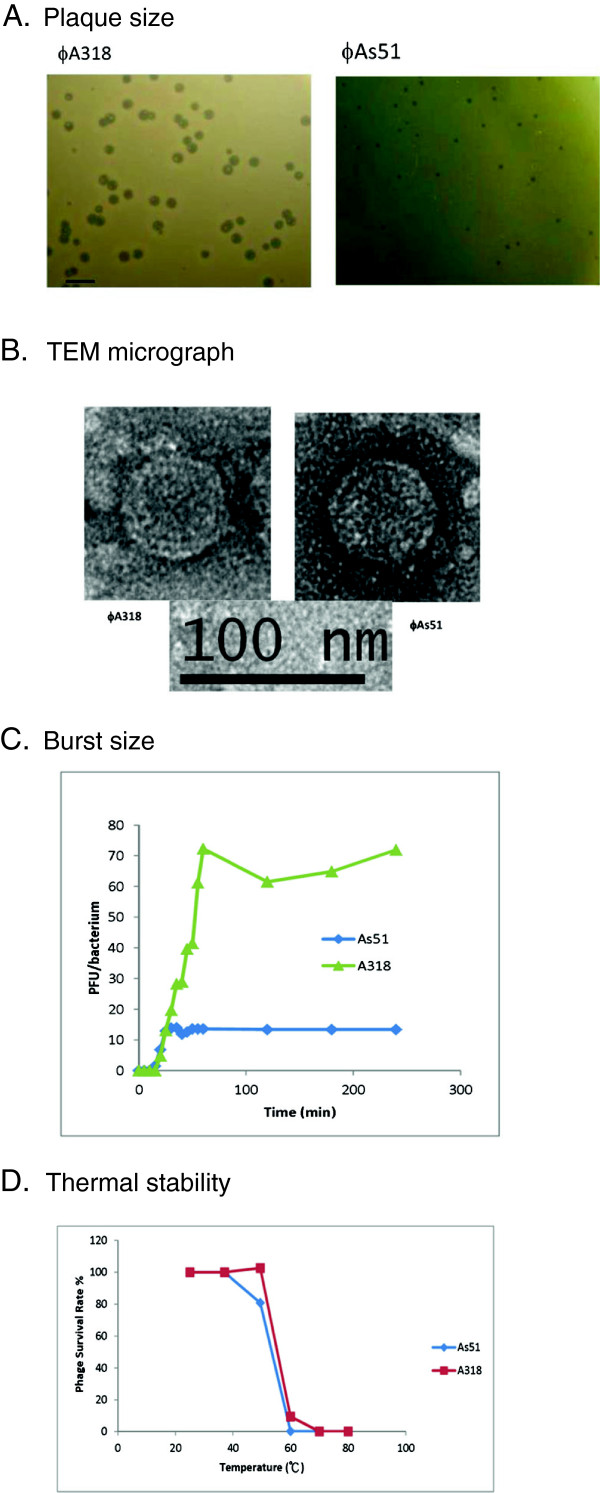
**Different characteristics between phages ϕA318 and ϕAs51. (A)** The plaque sizes within 16 h after phage infection at 25°C. **(B)** Transmission electron micrographs of phage particles. Virions were negatively stained with uranyl acetate. The bars represent a length of 100 nm. **(C)** One-step growth curves of phages in *V. alginolyticus* at 25°C for ϕA318 and 37°C for ϕAs51. The burst sizes were calculated by the amplifying ratio of Pt/P0 (phage titer at the plateau phase is divided by the inoculating phage titer). **(D)** Thermal stability of phages at various temperatures one hour. Samples were taken to determine the titer of the surviving particles and to calculate the percentage of infectious phage. In the stationary phase, standard errors (SE) for each phage were consistent at 16-20% (SE/mean), while the standard errors (SE) were approximate 16-72% (SE/mean) during the exponential phase.

The plaques of ϕA318 that formed in 4 hours at 25°C were very clear at their center and at the margin of the edge; sizes of the plaques were about 3 mm in diameter, which increased to 5 mm overnight (Figure 1A). The phage concentration in each plaque infected and fully lysed the amounts of host bacterium up to a 50x volume ratio. In comparison, the plaque size of ϕAs51 only reached 1 mm overnight (Figure 1A), and the amplification should not be greater than 3-5x dilution ratio. The EM morphology of phages ϕA318 and ϕAs51 was indistinguishable from each other. The icosahedral head was almost isometric, with a size of 50–55 nm, and the shells on the head capsids sketched out at least three visible rings, although the tail fiber was barely distinguishable from the capsid shell in the micrograph (Figure 1B). The tail was 12 nm long, elongating from 25 nm wide in the head-neck connector to 5 nm in the nozzle. They are highly similar to the bacteriophages of the family *Podoviridae* [7].

The susceptibility of other *Vibrio* strains to phage ϕA318 and ϕAs51 was also investigated by the agar overlay method. Among six other Vibrios, *V. damsela* and *V. harveyi* were found to be susceptible to both phages ϕA318 and ϕAs51, while the other four species could not be infected even at an MOI of more than 100. For measurement of the burst size, a bacterial culture in the early exponential growth phase was split into glass tubes with equal numbers of *V. alginolyticus* cells (MOI = 1.0), and then plaque titers were calculated within 4 hours of incubation. According to the one-step method [8], the burst size of phage ϕA318 was 72 PFU per infected cell, while that of ϕAs51 was 10 PFU/cell (Figure 1C).A thermal stability test was carried out to analyze the heat resistance of phages at pH 7.5-8.0. The phages were incubated at 25 - 80°C for one hour. As Figure 1D shows, the relative phage titers were measured at different temperatures. After incubation at 50°C for one hour, phage ϕA318 retained almost 100% infectivity, while ϕAs51 decreased to 80%. When the temperatures rose higher than 70°C, nearly all phages were inactivated after 15 minutes of incubation.

### The ϕA318 genome

**Figure 2:**
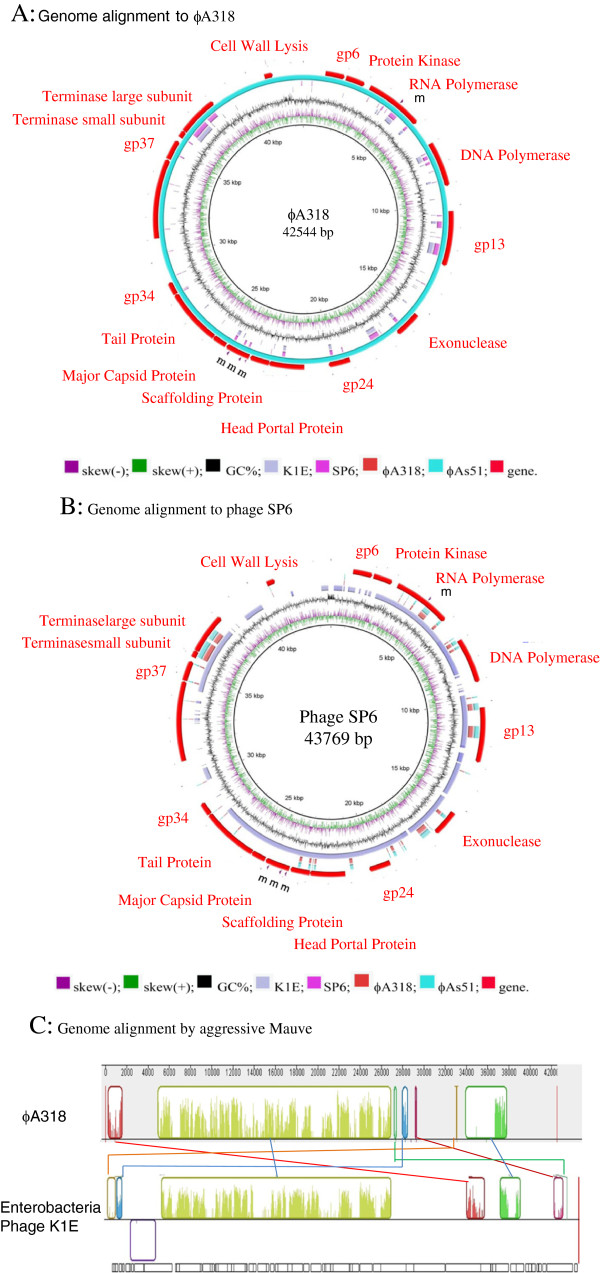
**Genome alignment and annotation for phages ϕA318 and ϕAs51 with the genomes from phages SP6 and K1E. (A)** Genome alignment using query of ϕA318 against to phages ϕAs51, SP6 and K1E, **(B)** Alignment using query of SP6 against phages K1E, ϕA318, and ϕAs51. ϕAs51 map is highly similar to ϕA318 with 5 single nucleotide mutations (marked by m) and annotation as shown in Table 1 and GenBank access no. KF322026 and KF800937. This draw was done by BLAST [9] and Ring Image Generator [32]. Purple line represents skew(-); green for skew(+); black for GC%; grey for K1E; pink for SP6; blur red for ϕA318; cyan for ϕAs51; red for specific genes. **(C)** Mauve was used to efficiently construct multiple genome alignments, which provides a basis for research into comparative genomics among phages ϕA318 to its related phage K1E.

ϕA318 genome was sequenced by a combination of shotgun sequencing (Figure 2 and Table 1). Open reading frames (ORFs) were determined using a combination of RAST visual inspection and translated tBLASTx searches [9]. The genome consists of a single double-stranded DNA molecule of 42544 bp with a GC content of 43.5%. We have annotated 47 open reading frames (The genome was submitted to GenBank with access no. KF322026 for phage ϕA318; [GenBank:KF322026]; Additional file 1: annotation for ϕA318 genome), all but one of which are transcribed on one strand of the DNA molecule. The ϕA318 genome is flanked by some terminal repeats. It is a tightly packed genome with ~90% of the sequence predicted to encode proteins. We use the same gene-numbering system by RAST PEG, starting from left to right on the genetic map, similar to that of K1E and SP6 since the genomes are analogous in organization of major predicted sequences (Figure 2A). ORFs that have no sequence similarity to any previously characterized *Podoviridae* phages are simply painted in gray (Figure 2B). Like phage K1E, as well as other T7-like phages, the ϕA318 genome can be divided into three regions: early, middle, and late (Table 1). The first predicted gene similar to other genomes is gp6, which is likely to be of unidentified function in SP6. Other than the endosialidase (see below), most open reading frames carried by ϕA318 are comparatively more similar to phages K1E and SP6 than to T7 and other known podoviruses that infect *Vibrio*. ϕA318 encodes an RNA polymerase (RNAP of PEG 5; gene 1.0 or K1Ep10) which is responsible for transcription of most of other phage genes that are involved in DNA replication, maturation, and packaging [10]. An adenylation DNA ligase-like gene (PEG 24, ~gene 1.3) is analogous to that of SP6p25 or K1-5 gp24 and is present at the end of the Class II genes. The middle region encodes mainly proteins involved in DNA replication/metabolism, among which only primase/helicase (PEG 9, gene 4.0), DNA polymerase (DNAP; PEG 12, gene 5.0), and exonuclease (PEG 19, gene 6.0) were annotated functionally.

**Table 1:** **The genes in genomes of phages ϕA318 and ϕAs51 were annotated to known functions**

Class	T7	K1E	ϕA318 ORF	Start	End	Function	Aliases
I	gene 0.3?		3	1023	1916	gp6 protein	Inhibits *Eco*B and *Eco*K host restriction
I	gene 0.7		4	1990	2886	protein kinase	
I	gene 1	gene 7	5	3173	5957	RNA polymerase*	ϕAs51: 3173..4727 and 4615..5955
II	gene 4A	gene 9	9	6997	9093	DNA primase	
II	gene 5		12	10263	12806	gp13	Putative DNA polymerase
II	gene 6		19	16306	16599	hypothetical protein	Era10.24 Phage exonuclease 40.13
I	gene 1.3		24	19109	20107	gp24	SP6p25 Adenylation_DNA_ligase_like; K1-5 gp24
III	gene 8	gene 29	29	21233	22861	head portal protein	
III	gene 9	gene 30	30	22861	23769	scaffolding protein	
III	gene 10B	gene 31	31	23817	24968	major capsid protein*	
III	gene 11	gene 32	32	25042	25689	tail protein	
III	gene 12	gene 33	33	25692	28313	tail protein	
		gene 34	34	28323	28928	gp34	K1E gp34 internal protein
III	gene 13	gene 36	36	31035	34724	internal virion protein	
III	gene 17	gene 37	37	34759	35694	gp37	K1E endosialidase adaptor protein; SP6gp38 tail fiber/adaptor
III	gene 18	gene 39	39	35889	36233	hypothetical protein	Era10.45 Phage terminase, small subunit 33.01
III	gene 19	gene 40	40	36233	38107	large terminase subunit	
			44	40747	41136	similar to cell wall hydrolyses	
						involved in spore germination	

*Five nucleotide mutations were found in ϕAs51, which can be referred to text.

With the high similarity to other phages, the late region of the ϕA318 encodes the virion structural proteins as well as many of the proteins involved in maturation and cell lysis. The ϕA318 PEG 29 is the head-tail connector (gene 8.0), followed by those coding for the scaffolding protein (gene 9.0), capsid (gene 10.0), tail tube (genes 11.0 and 12.0), and internal virion proteins (gene 13.0-16.0). The genes coding for maturation and terminases subunits (genes 18 and 19.0) were found in PEGs 39 and 40. Perhaps the PEG 37 (~gene 17) coded for the product with only a small region of amino acid similarity to the T7 tail fiber at the N-terminal head-binding portion, whereas the central catalytic region is highly similar to that of phage K1E endosialidase which may be involved in both recognition and depolymerization of the K1 polysaccharide capsule.

**Figure 3:**
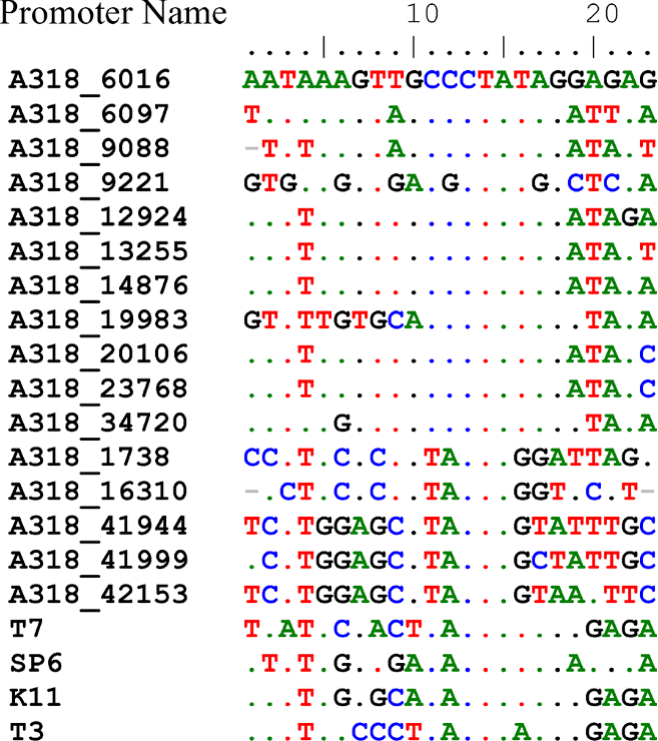
**The sixteen promoters in phage ϕA318 genome were predicted and aligned with those of T7, SP6, K11, and T3 phages.** The position of each promoter in ϕA318 genome was recorded as the number following the name of phage. The names of predicted promoters are given using their positions in ϕA318 genome, while the positions are shifted by two nucleotides in ϕAs51 genome.

Like most of members of the closely-related T7 family, ϕA318 is flanked by terminal repeats, suggesting similar replication strategies: phiL-122, 124, and 126 were promoter-like and repeats. Promoter sequences for most gene transcription among T7 supergroup phages, which are driven by phage-encoded RNAPs, are recognized very specifically. ϕA318 encodes an RNAP with highly conserved motifs to that of T7. Employing the UGENE program for searching at repeats with the conditions of >13 bases and >95% homology, we predicted 16 promoter consensus sequences from 126 repeat regions: 11 putatively strong promoters and six weak ones. Most of those have T7-like homologues (Figure 3). The consensus sequence from −7 to +1 is identical to that of T7, except that A at −6 was replaced by C. However, bases of −8 through −12, which are important for promoter specificity, differ from T7, T3, K11, and SP6 [10, 11]. The consensus at −9 for T7 and SP6 is C, any change leading to inactive [10]. Since ϕA318 and K1E have T and G, respectively, at this position, it is unlikely that the T7 or K1E RNA polymerase will initiate transcription from ϕA318 promoters. ϕA318 RNAP has different promoter specificity.

ϕA318 appears to have phage promoters throughout the region of the T7 analogues. We proposed that the promoters of phi122, phi124, and phi125 in ϕA318 are equivalent to φOR involved in packaging and possibly replication [10]. Unlike phages gh-1 and K1E, one phage-specific promoter (phi-A8 at position 1741) was found immediately upstream of the protein kinase and RNA polymerase genes in ϕA318, suggesting that the autoregulation controls these two genes together. The genome contains a strong σ70 promoter (at 229–257 nt) responsible for early gene transcription, including phage RNA polymerase. The end of transcription of the early genes in ϕA318 is marked by **GCCCTGAT**tcttaatgag**TCAGGGC**TTTT CTT, a rho-independent terminator (TE, near position 5957) immediately following the RNA polymerase gene, the equivalent position at 7128 to 7161 in K1F of such terminator. PEG 4 in ϕA318 is similar to gene 0.7 in T7, which could encode a kinase to phosphorylate host RNAP and efficiently inactivate the host gene expression [12]. Two terminators, **GCCCCTGCC**tacttt**GGTAGGGGC**TTATTTTT at position 24971 and **GAGGGACT** cctaag**AGTCCCTC**cTTTCTT at position 38217, were found. Terminator at 24871 was a potential analogue to the T7 Tφ terminator, located inside the gene encoding major capsid; terminator at 38217 was just the end of tail fibers.

### Genome comparisons between ϕA318 and ϕAs51

Analyses of nucleotide restriction enzyme polymorphisms and protein profiles showed no difference between ϕA318 and ϕAs51 (data not shown); the amplification rates were, however, significantly distinct for the two phages. It was worth-while to sequence the whole genomes to investigate the context. ϕAs51 genome of 42542 bp (GenBank access no. KF800937; [GenBank:KF800937]; Additional file 2: annotation for ϕAs51 genome) was almost the same as ϕA318, except that five single nucleotides were mutated sporadically in the genome. Triple checks by PCRs and re-sequencings showed that two deletions of Adenosine were in RNAP gene while single-nucleotide mutations in three sites also occurred in the major capsid protein.

**Figure 4:**
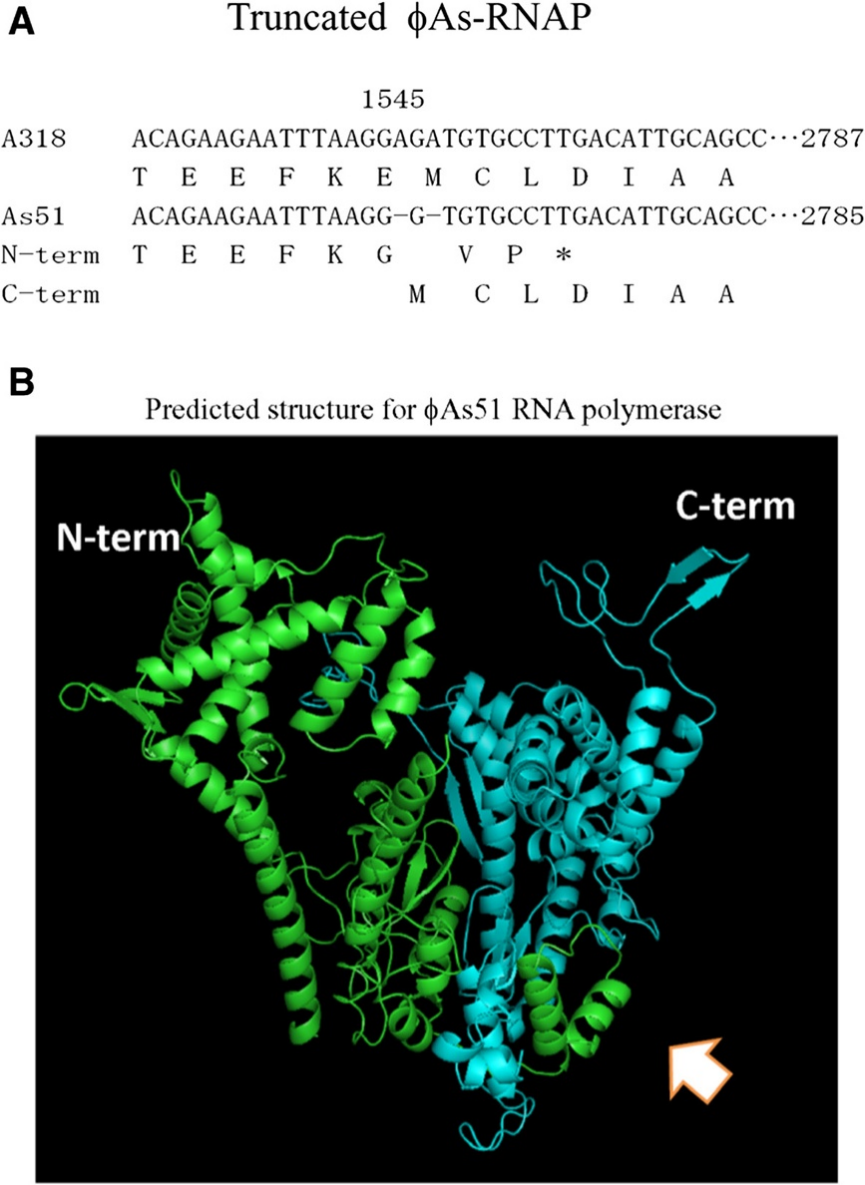
**Location of nucleotide deletion in ϕs51 and proposed products of subunits. (A)** Two nucleotides were deleted found after 1545 nt in ϕAs RNAP. The proposed new ribosomal binding site was underlined. **(B)** Two fragments of peptides were predicted by PHYRE^2^ for 3D structures. Green represents the N-term of full RNAP, while the cyan is for C-term. The predicted structures were superimposed together onto T7 RNAP skeleton.

In comparison with gene sequences of other T7-like RNA polymerases, ϕA318 and ϕAs51 RNAP genes shared five consensus regions, corresponding to residues 421–425, 537–538, 563–575, 627–641, and 811–813 residues in the T7 RNA polymerase. As shown in our previous publication [7], the motifs were highly conserved among the T7-like phages. In phage ϕA318, the sequences of the motifs were DFRGR (motif T7-421), DG (motif T7-537), PSEKPQDIYGAVS (motif T7-563), RSMTKKPVMTLPYGS (motif T7-627), and HDS (motif T7-811), respectively. In the region of ϕAs51 RNAP gene, two nucleotides were deleted at 1545 nt, resulting in that the RNA polymerase molecules were translated into two pieces: one N-term fragment of the 515 residues plus three additional residues and the second fragment for C-term with just one additional Met as a start codon (Figure 4A). The analysis of nucleotide sequence revealed that the ribosome binding site AGAAGAAT upstream of a GTG was suitable for the second translation of ϕAs51 RNAP gene to a product of the exactly same sequence as the C-term of ϕA318 RNAP. With use of the threading method of PHYRE^**2**^ to predict the 3-dimentional structures for these two fragments of ϕAs51 RNAP, the result revealed that the truncation join was located in the junction of palm and fingers. As Figure 4B shows, the tail of ϕAs51 RNAP N-term fragment formed a clip (arrows) to hold the ϕAs51 RNAP C-term fragment at the position on palm.

**Figure 5:**
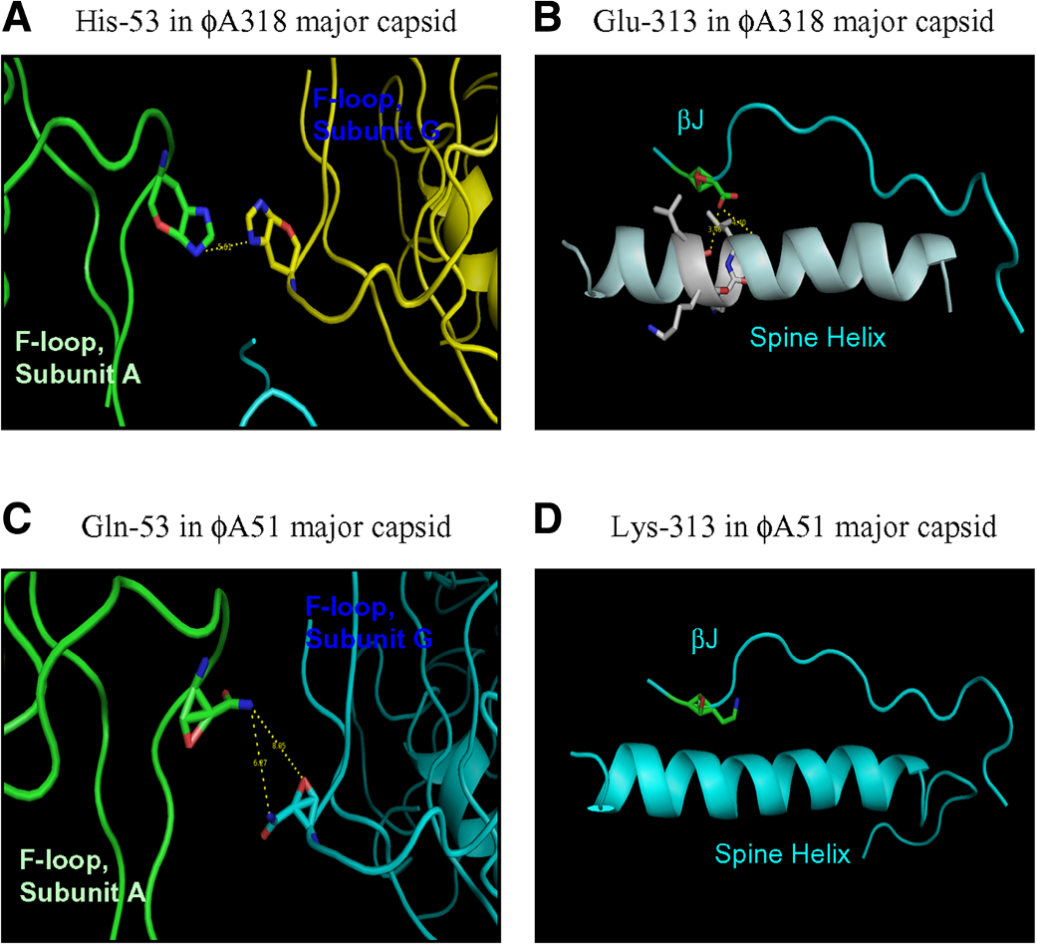
**Point mutations found in ϕAs51 and losses of interaction in the predicted structures of subunits.** Dot lines between atoms represent the distances. βJ is located in P-domain. The pair of His-53 is in the F-loops of two capsomere subunits or between the subunit G and neighboring hexamer.

Three mutations of single nucleotide were all in the first codon of the residue, and therefore, totally altered the residues. The mutation at 23973th nucleotide resulted in mutating Histidine at 53th residue to be Asparagine (H53N); 24171th nucleotide mutation changed Isoleucine at 119^th^ to Valine (I119V); and 245789^th^ nucleotide mutation converted Glu-313 to Lys-313 (E313K). The structures of ϕA318 and ϕAs51 major capsid proteins were predicted from models of HK97 and T7 capsids by PHYRE^2^ with >93% confidence. In comparing with the ϕA318 capsid, the mutation effects of Asn-53 on ϕAs51 major capsid protein broke the presumed cation bridge between His-53 of two capsomere subunits in ϕA318 capsid (Figure 5A and C). Glu-313 residue of βJ in P-domain formed two hydrogen bonds with the O = C of the backbone of the Spine Helix to stabilize the link between three beta-sheets and one helix (Figure 5B). However, E313K mutation in ϕAs51 caused the βJ in P-domain to lose two hydrogen bonds so as to destabilize the link on the Spine Helix (Figure 5D).

## Discussion

This is the first *Podoviridae* genomes of *Vibrio alginolyticus* phages that are documented in GenBank. 16 out of 47 ORFs were annotated to known functions, while other ORFs were similar to enterobacteria phages K1E and SP6. 11 strong phage promoters and three terminators were predicted, which are located slightly differently from the closest partners, though genomic organizations were highly homologous [10, 12]. Most of promoters have T7-like homologues, except that A at −6 was replaced by C and specificity region on −8 to −12 (Figure 3). In ϕA318 and ϕAs51 phage promoters, the consensus sequence from −7 to +1 is **CCCTATAG**. However, bases of −8 through −12, which are important for promoter specificity, differ from T7, T3, K11, and SP6. The consensus at −9 for T7 and SP6 is C; any change of this nucleotide inactivates the promoter function [10]. Since ϕA318 and K1E have T and G, respectively, at this position, it is unlikely that the T7 or K1E RNA polymerase will initiate transcription from ϕA318 promoters. ϕA318 RNAP has different promoter specificity. This exclusivity of promoter specificity seems to be the trend among the T7-like phages, and it seems that there must be some selective pressure that resulted in this feature.

Electron microscopy revealed that phage ϕA318 and ϕAs51 particles were morphologically similar to type C in Bradley’s classification of *Podoviridae* phage (Figure 1) [13]. The podovirus family is characterized by phages with a nearly isometric icosahedron head and a short non-contractile tail [13, 14]. Furthermore, the lengths of the icosahedral capsid and tail of phage ϕA318 were morphologically indistinguishable from those typically observed for ϕAs51.In phages ϕA318 and ϕAs51, the sequences of the motifs were DFRGR (motif T7-421), DG (motif T7-537), PSEKPQDIYGAVS (motif T7-563), RSMTKKPVMTLPYGS (motif T7-627), and HDS (motif T7-811), respectively. Strikingly, two nucleotides were deleted in the region of ϕAs51 RNAP gene, which resulted in the RNA polymerase molecules being translated into two pieces separated at the 515th residues (1545 nt); the N-term fragment increased three additional residues in its C-terminus while the second fragment started from a suitable upstream ribosome binding site AGAAGAAT and added just one codon GTG for Met to form exactly the same product as the C-term of ϕA318 RNAP (Figure 4A). We suggested that the two fragments could form a 2-subunit-like enzyme to function for the RNA polymerization but weaken RNAP and lose 88% bursting ability.

Virus maturation corresponds to a transition from an initial non-infectious to an infectious and robust virion. PHYRE2 matched the major capsid protein of ϕA318 and ϕAs51 to phage T7 capsid structure PDB:3IZG [15, 16]. The 3-dimentional prediction clearly revealed that the capsids of the two new phages were characterized by A-domain, Spine Helix, P-domain, E- and F-loops. Three β-strands are located in P-domain, where βJ is in the center of the three. The matches of residues, however, were not by one-on-one basis. The 53th residue of ϕA318 and ϕAs51 capsid was ruled onto 156^th^ residue in F-loop of PDB:3IZG while 115-135th residues of ϕA318 and ϕAs51 were corresponding to 170-190th residues of Spine Helix in PDB:3IZG. The prediction clearly showed Spine Helix and βJ is located in P-domain. The pair of His53 is in the F-loops of two capsomere subunits or between the subunit G and hexamers. A fusion of the scaffolding protein to be δ-domain, residues 2–103, in HK97 capsid was not found in the new phages, suggesting that the dynamic of forming matured capsid of ϕA318 and ϕAs51 may differ from HK97. Based upon our predicted capsid that was well aligned with the structure of residues 104–385 in the HK97 coat subunits, which form a mixture of hexameric and pentameric capsomeres upon expression, they may still share some degree of expansion intermediates with the HK97 capsid [17]. For example, 415 coat subunits (60 hexamers and 11 pentamers) assemble with a dodecameric portal and ~60 copies of the viral protease to form proheads; or the prohead particles exhibit spine helix bent and P-domain β-sheet twist. Veesler *et al.* [17] used an HK97 subunit mutation that prevents formation of non-covalent interactions in virus-like particles and stops maturation at the expansion intermediate of the particles without E-loop “chainmail” interactions. Our results of ϕAs51 also showed E313K in βJ may be involved in the spine helix bending and P-domain β-sheet twisting, while H53N mutation may prevent non-covalent interactions of E-loop chainmail. When Histidine at 53th is present in the F-loop, adjacent to E-loop, divalent cations like Ni^2+^ and Ca^2+^ will build a bridge for such interactions.

Capsid instability in ϕAs51 with residues Asn-53 (53 N) and Lys-313 (313 K) can account for the thermal stability data in Figure 1. We proposed that the deletion effects of RNAP contributed higher percentages of losing burst ability for ϕAs51 phage than the point mutations occurring in the major capsid protein. Our findings suggest that the genome sequencing provides a security check prior to application of phages in therapy; thereafter, the mutation rate for each hotspot can be validated for managing the medication plan. The complicate mechanisms still lie ahead for us to solve. Once phage decreases gene activities, the host gains a niche to suppress the phage propagation; as a seesaw does [11]. Further investigations will discover the details.

## Conclusions

Five nucleotide mutations sporadically spread in the ϕAs51 genome of about 42500 nucleotides. Those small differences may be the causes to distinguish the lower propagation ability and thermal tolerance of ϕAs51 from the superior ϕA318 in plaque size. Subtle analyses on the residue alterations uncovered the effects of five nucleotide mutations on the functions of the RNAP and capsid proteins, which account for the host-bursting efficiency. The deletion of two nucleotides in RNAP gene truncates the primary translation due to early stop codon, while a second translational peptide starting from GTG just at deletion point can remediate the polymerase activity. Out of three mutations in major capsid gene, H53N mutation weakens the subunit assembly between capsomeres for the phage head; E313K reduces the fold binding between β-sheet and Spine Helix inside the peptide. Genome sequencing has been done for phage therapy and probiotics to detect a tiny mutation but great effects which can provide early prediction of treatment efficacy or potential side effects.

## Methods

### Bacterial strains and growth conditions

*Vibrio* strains were bought from the Bioresource Collection and Research Center, Taiwan, including *V. alginolyticus* ATCC 17749, *V. carchariae* ATCC 35084, *V. damsela* ATCC 33536, *V. harveyi* ATCC 14126, *V. parahaemolyticus* ATCC 17802, *V. pelagius* ATCC 25916, and *V. vulnificus* BCRC15431. The *Vibrio* strains were maintained in Bacto Brain Heart Infusion medium (BHI; Becton-Dickinson Co, USA) supplemented with 3% NaCl (Panreac Quimica, France). For long-term preservation, bacteria were frozen at −80°C in BHI supplemented with 1% NaCl and 25% glycerol (Nihon Shiyaku, Japan). The strains were streaked onto modified seawater yeast extract (rich MSWYE) agar plates consisting of 23.4 g NaCl, 6.98 g MgSO_4__7 H_2_O, and 0.75 g KCl in 1000 ml distilled water [18]. The pH was adjusted to 7.6 with 1 N NaOH, followed by the addition of 5.0 g of proteose peptone (HIMEDIA Lab, India), 3.0 g of yeast extract (HIMEDIA Lab, India), and 20.0 g of agar per liter.

### Isolation and titer of bacteriophages

Water samples were collected from aquaculture waterways in southern Taiwan between 2008 and 2010. The water was centrifuged at 10,000 × *g* for 30 minutes, and the supernatants were filtered through a 0.45 μm microfilter. After centrifugation and microfiltration, 20% MSWYE medium [7, 8] and 1% overnight-grown *Vibrio alginolyticus* were added to the filtrate and incubated at 25°C for 4–24 hours to enrich the phage growth. The bacterial debris was removed by two centrifugations at 10,000 × *g* for 30 minutes. After enriching the phages in the field samples with the target bacterium *Vibrio alginolyticus* for 4–16 hours, phage-containing supernatants were incubated with the indicator strain for 5 minutes, suspended in low-percentage agar (top agar), and plated onto solid agar plates, so the target strain would form a bacterial lawn with the formation of plaques. The ten largest plaques from each plate were picked and re-amplified three times in bacterium-containing broth using a 5× volume ratio. The plaques were picked into 200 μl MSWYE broth for further titer counting or production amplification. To prepare bacterial cells for determination of phage concentrations, the host *Vibrio alginolyticus* was freshly inoculated as a 1% volume of seed from overnight culture into 10 ml of rich MSWYE broth, and it grew to an OD_600_ of 0.3-0.4 in about 2 hours. After a series of dilutions, 10 μl of diluted phage were added to 200 μl of bacterial cells, incubated for 5 minutes, mixed with 3 ml of top agar (rich MSWYE with 0.5% agar), and then poured onto the solid surface of a 2% agar plate. The plaques were counted in 3–5 hours; the titer per ml was calculated as 100 × (dilution factor) × (plaque count).

### Electron microscopy

Preparation of phage particles for electron microscopy has been described elsewhere [19]. In brief, bacteriophage particles were applied to parafilm to produce a spherical drop. Carbon-coated nitrocellulose films were fabricated on copper grids, which were placed face down on the sample drop for 1 min to absorb the particles. After being briefly washed twice in 10 μl of 10 mM Tris buffer (pH 8) (Amresco, USA), the samples were then rinsed twice with freshly prepared 2% uranyl acetate (UA; Sigma-Aldrich, USA) in Tris–HCl (pH 8.0) and stained for 60 seconds. Following each step of absorption, washing, and UA staining, the grids were blotted with filter paper until almost dry. The finished grids were dried in vacuo overnight. Images of phage particles were taken at a magnification of 40,000× and a defocus of 3 μm, using a 200-kV electron microscope (JEOL JEM-2010, equipped with a Gatan-832 CCD camera).

### DNA Preparation for bacteriophage

For propagation of phage, 10 ml of phage stock was added to 100 ml of *V. alginolyticus* (3 × 10^8^ CFU ml^−1^) cultured in MSWYE and incubated in a shaker at 25°C for 3–5 hours until the lysate was clear. The remaining cells and debris were removed by two centrifugation cycles at 10,000 × *g* for 30 minutes. The supernatant, with a titer of 2 × 10^10^ PFU ml^−1^, was stored at 4°C as a phage stock. To concentrate phages using a standard PEG protocol [7, 20], solid NaCl and polyethylene glycol 8000 (Fluka, Germany) were added, and precipitation was performed overnight at 4°C. After centrifugation, the phage particles were resuspended in 2 ml of SM buffer and treated with DNase I and RNase A (Sigma, USA) to remove contaminating nucleic acids from the host. The polyethylene glycol was extracted by adding an equal volume of chloroform (TEDIA, USA) until the interface was clear. The aqueous phase containing the phage was treated with proteinase K (Invitrogen, USA) and sodium dodecyl sulfate (SDS; MD Bio, Inc., USA) at 56°C for 1 h. Phenol extraction was carried out three times at room temperature, and the aqueous phase was further extracted with a 1:1 mixture of equilibrated phenol (Sigma, USA) and chloroform. DNA precipitated by a 2x volume of cold ethanol was redissolved in deionized water.

### Adsorption and phage burst size

As described previously [7, 8] for the adsorption of phage on bacteria, 1 ml of broth containing host cells and phages was taken at the 5th and 10th minutes and centrifuged to remove bacteria and bound phage, and free phage in the supernatants was then measured. Similarly, the burst size was measured as described previously [7]. In brief, *V. alginolyticus* cells were grown in 20 ml of medium to mid-exponential phase (OD_600_ = 0.3-0.5) and mixed with 0.1 ml phage solution (MOI = 1). Samples of 1 ml of the mixture were taken at intervals and immediately subjected to centrifugation at 14,000 × *g* for 3 minutes to remove bacteria. The phage titers in the solutions were determined by the agar overlay technique. Using the growth curve for MOI = 1 according to the one-step criteria, the burst size (Bs) of phage was calculated as Bs = Pt/P0, where Pt is the phage titer at that plateau phase and P0 is the initial infective titer.

#### Genome sequencing and annotation

Similar to the shotgun sequencing described elsewhere, approximately 5 μg of the bacteriophage genomic DNA was randomly sheared by nebulization, and DNA sequencing was performed at Mission Biotech according to the manufacturer’s protocol for the Genome Sequencer GS Junior System (Roche Diagnostic). Low quality sequences of the reads generated by the GS Junior sequencer were trimmed off. *De novo* assembly of the shotgun reads was performed with the GS Assembler software. Sequence assembly and analyses were performed essentially as previously described. Protein-encoding genes (PEG) were predicted using The RAST Server (Rapid Annotations using Subsystems Technology; http://rast.nmpdr.org/) [21] and analyzed with the SEED-Viewer (http://www.theseed.org/wiki/Main_Page) [22]. Protein-coding genes were also checked using the *ab initio* gene-finding program Glimmer v3.02 [23]. rRNA and tRNA genes of the draft assembly were identified using RNAmmer [24] and tRNAscan-SE [25]. Automatic functional annotation results obtained by the RAST were further compared with the proteins in the GenBank database using PSI-BLAST (http://www.ncbi.nlm.nih.gov/blast/Blast.cgi). The repeat units in the phage genomes were found by UGENE tool [26] and further aligned to acquire the consensus regions. The phage rho-independent terminators were predicted by ARNold with the criteria of both Erpin and RNAmotif selections [27]. The genomes of ϕA318 and ϕAs51 were submitted to GenBank with access no. KF322026 and KF800937, respectively [GenBank:KF322026; GenBank:KF800937].

### Multiple sequence alignment

To determine the classification status of the newly isolated ϕA318 and ϕAs51 phages, sequence data for the genes encoding the DNA polymerase, DNA ligase, RNA polymerase, single-strand DNA binding protein, and capsid proteins of enterobacteria phages (T7, SP6, K1E, and K1F) and *Vibrio cholerae* phages (N4, VP4, and ICP3) were employed to find highly homologous regions. Complete genome sequences of four enterobacteria phages and three *Vibrio Podoviridae* phages were acquired from National Center for Biotechnology Information, NIH, USA: T7 (39,937 bp) [GenBank:NC_001604], SP6 (43,769 bp) [GenBank:NC_004831], K1E (45,251 bp) [GenBank:NC_007637], K1F (39,704 bp) [GenBank:NC_007456], ICP3 (39,162 bp) [GenBank:NC_015159], N4 (38,497 bp) [GenBank:NC_013651], and VP4 (39,503 bp) [GenBank:NC_007149]. The T7 RNA polymerase sequence is also the same as PDB:1QLN (RCSB-PDB ID), and SP6 RNA polymerase is from NC_004831 [GenBank:NC_004831]. The threading predictions by PHYRE ver2 [15] were iterated to predict the tertiary structures of ϕA318 and ϕAs51 RNA polymerase.

Sequences of individual genes which were retrieved from the genome sets were then aligned using ClustalW with default options [28]. The complete RNA polymerase sequences were analyzed by the neighbor-joining method using the NEIGHBOR program in Phylogeny Inference Package (PHYLIP) [29]. Distances were calculated using the DNADIST program of PHYLIP and displayed in TreeView. ClustalW, PHYLIP, and TreeView were bundled in the BioEdit program version 7.0.5 [30]. The multiple sequence alignment for the phage genomes was constructed in the Mauve using MUSCLE3.6 algorithm [31].

## Electronic supplementary material

Additional file 1: **phiA318.gbk.** It is in GenBank format. Annotation for ϕA318 genome. (ZIP 29 KB)

Additional file 2: **phiAs51.gbk.** It is in GenBank format. Annotation for ϕAs51 genome. (ZIP 30 KB)
